# The StrongWomen Change Clubs: Engaging Residents to Catalyze Positive Change in Food and Physical Activity Environments

**DOI:** 10.1155/2014/162403

**Published:** 2014-11-30

**Authors:** Rebecca A. Seguin, Sara C. Folta, Mackenzie Sehlke, Miriam E. Nelson, Eleanor Heidkamp-Young, Mark Fenton, Bridgid Junot

**Affiliations:** ^1^Division of Nutritional Sciences, Cornell University, Ithaca, NY 14850, USA; ^2^Friedman School of Nutrition Science and Policy, Tufts University, Boston, MA 02111, USA; ^3^Boston Public Schools, Boston, MA 02111, USA; ^4^Athletics House, Level 2, 31 Aughtie Drive, Albert Park, VIC 3206, Australia

## Abstract

*Introduction.* The epidemic of obesity is a multifaceted public health issue. Positive policy and environmental changes are needed to support healthier eating and increased physical activity. *Methods.* StrongWomen Change Clubs (SWCCs) were developed through an academic-community research partnership between researchers at Cornell University and Tufts University and community partners (cooperative extension educators) in rural towns in seven U.S. states. Extension educators served as the local leader and each recruited 10–15 residents to undertake a project to improve some aspect of the nutrition or physical activity environment. Most residents had limited (or no) experience in civic engagement. At 6 and 12 months after implementation, the research team conducted key informant interviews with SWCC leaders to capture their perceptions of program process, benchmark achievement, and self-efficacy. *Results.* At 12 months, each SWCC had accomplished one benchmark; the majority had completed three or more benchmarks. They described common processes for achieving benchmarks such as building relationships and leveraging stakeholder partnerships. Barriers to benchmark achievement included busy schedules and resistance to and slow pace of change. *Conclusion.* Findings suggest that community change initiatives that involve stakeholders, build upon existing activities and organizational resources, and establish feasible timelines and goals can successfully catalyze environmental change.

## 1. Introduction

Obesity is understood to be the result of complex socioecological factors and interventions that target multiple levels of the socioecological framework, including individual, social, built environment, and policy factors, are more likely to be successful [1–3].

Civic engagement is one approach to create support for environmental change. However, the effectiveness of civic engagement projects and/or coalition work to address obesity is difficult to measure, primarily because goals and activities differ between communities [4]. In a comprehensive review examining community coalitions focused on a range of health promotion initiatives, Zakocs and Edwards report inconsistent results when examining factors associated with coalition effectiveness; some factors associated with success were group cohesion, leadership, agency collaboration, member participation, and diversity [5]. Similarly, Roussos and Fawcett reported that while findings are insufficient to make strong conclusions, community-level coalitions aimed at changing systems and/or behavior hold potential in affecting population-level health outcomes [6]. Other research indicates that individuals engaged in community coalitions perceived benefits including improved physical fitness, nutrition, self-confidence, self-esteem, sense of personal empowerment, and social relationships [7]. Overall, the literature suggests that civic engagement is appropriate for initiating changes that can improve community health, but there is still much to be learned.

The socioecological model was the foundational theory for the design of the StrongWomen Change Club (SWCC) project's curriculum which aimed to engage individuals to identify an issue relevant to them in their community context and facilitate implementation of an action plan that would affect social, cultural, environmental, and political factors within each of their seven communities. The aim of this paper is to describe the processes by which the research team (RAS, SCF, MS, MEN, EY, MF, and BJ) engaged community partners (each SWCC and its leader) to improve their nutrition and physical activity environments. We provide a starting point for understanding best practices related to civic engagement as a means of creating communities in which the healthy choice is the easier choice.

## 2. Methods

This study was derived from a partnership of approximately ten years of collaboration between researchers at Tufts University's StrongWomen Program (SWP) and community health educators/leaders with the National Institute of Food and Agriculture (NIFA) Cooperative Extension Programs [8–11]. The research team designed the SWCC project and supporting curriculum as a vehicle for an academic-community civic engagement partnership focused on creating local change in active living and healthy eating.

The research team partnered with NIFA cooperative extension educators, who are active and well-connected local leaders, to most effectively identify both relevant community issues and to realistically assess community resources to best facilitate interventions [12]. We chose existing StrongWomen exercise groups as the partner for the SWCC initiative because they demonstrated a baseline level of interest and readiness to engage in issues of community health and wellness.

To identify the seven communities, we worked with state level StrongWomen Ambassadors and focused on finding small communities (ideally, fewer than 15,000 residents) where there was a StrongWomen leader (all cooperative extension agents) who would be interested and willing to serve as a SWCC project leader. Once identified, we corresponded with these leaders over several months to prepare for in-person workshops by outlining expectations, starting to discuss the community's particular challenges, identifying potential community stakeholders, and providing guidance on formation of the “Change Clubs.”

The SWCC was defined as a group of approximately 8–15 committed community members, often current and former SWP participants, who were interested in undertaking a project to create positive environmental change relevant to nutrition or physical activity within their respective communities.

### 2.1. Approach

Study procedures were reviewed and approved by the Institutional Review Boards at Fred Hutchinson Cancer Research Center and Tufts University (note: RAS was formerly of Tufts University and is now at Cornell University). Written consent for the survey was obtained by SWCC leaders and participants at the in-person workshop; oral consent was obtained by phone during follow-up interviews. Individuals did not receive a research stipend/participant fee, but each SWCC group was provided $1,000 to help support their group's identified issue of focus.

Study activities took place September 2011–December 2012. As a first step, leaders received a formal introduction packet that outlined the roles and responsibilities of the leader, the change club rationale and purpose, tentative timeline and schedule, and sample agenda for the three-day workshop that would be conducted at each site. A second step was a “get to know your town interview” conducted via telephone by a research team member (MEN, EY, and BJ), designed to illuminate potential barriers and facilitators to healthy eating and/or physical activity within each town, including features such as the presence or lack of sidewalks and crosswalks or the availability of fast food versus fresh produce. SWCC leaders also received a packet of information on recruiting members, holding interviews with community stakeholders, holding the first meeting, and choosing their area of change/focus.

For a period of eight weeks, two or more members of the research team (RS, EY, MEN, and SF) traveled to seven small communities located in Alaska, Arkansas, Kansas, Missouri, Montana, Pennsylvania, and Wisconsin. The research team facilitated interactive workshops with the SWCC leader and all participants in each community that lasted three to four days. The researchers' primary role during these visits was to facilitate a structured planning and visioning process and guide team building exercises with the newly formed SWCC.

During the workshops, researchers provided educational materials and conducted awareness activities focusing on food and physical activity environments, personal habits, and potential areas for improvement. Each SWCC defined an overarching project goal (called a Noble Purpose), identified strategic community stakeholders, and developed action steps. Once this visioning process was complete, researchers assisted SWCC members in establishing specific, measurable benchmarks for goal attainment in the coming year. The SWCC curriculum, developed by the research team, guided each step in the process.

The year following the workshops represented the implementation and follow-up period for the SWCCs, during which the research team was minimally involved in the interventions. Researchers conducted brief monthly calls with the SWCC leader over six months to provide guidance and problem solving as necessary.

The first round of outcome assessments began six months after the workshop visit. A research coordinator conducted an hour-long key informant interview with the SWCC leader. The purpose of the interview was to (1) gauge the groups' progress on individual quantitative benchmarks, (2) understand any unintended consequences or ripple effects in the community as part of this project, and (3) to assess the leaders' self-efficacy and feelings of empowerment as community change agents as a result of this project.

The second round of outcome assessments was conducted at 12 months post-visit, again using telephone interviews. In addition to being asked to rate progress on each benchmark using a 5-point scale ranging from “did not accomplish” to “successfully accomplished,” these interviews also captured more extensive qualitative data on leaders' perceptions of the implementation process, overall perceptions of success, and facilitators and barriers to achievement.

By way of summary, the interview guide's primary topics and example questions were as follows.

*(i) Achievement of Benchmark(s)*

*Example Question*. In your opinion, has the Change Club accomplished (this) benchmark? 

*(ii) Factors That Contributed to Successful Benchmark Achievement*

*Example Question*. Please describe how your Change Club went about achieving this benchmark. 

*(iii) Barriers to Benchmark Achievement*

*Example Question*. What have been the major barriers that your Change Club has experienced when engaging in this type of work? 

*(iv) Individual Achievements Resulting from Participation in Change Club*

*Example Question*. Please tell me about any other activities or actions (ripple effects) that you can think of that have happened since we left your town? 

*(v) Leader's Perception of Ability to Effect Change Using the Change Club Model*

*Example Question*. On a scale of 0 to 10, where 0 to 2 stand for none or a little and 8 to 10 stand for a great amount, to what extent do you consider yourself a change agent role model for others?

### 2.2. Analysis

Qualitative data analysis was conducted by SF and MS between May and October 2013. Transcript files were entered and coded using NVivo (QSR International, version 9.0, 2011), led by MS with a subset of double coding and agreement checks between MS and SF. Manifest content analysis was used to analyze the data, employing the following process: verbatim transcription; transcription review; development of and connection between codes and themes (based upon the main interview topics); and data interpretation and review by the research team in an iterative process. Quantitative (survey) data were analyzed using SPSS version 21.

## 3. Results

Each SWCC initially included 8–15 female participants (*N* = 107), mean age 59 years, SD 14 (range = 22–85 years). Demographic characteristics of SWCC participants are shown in Table 1. Each SWCC collaboratively identified a Noble Purpose goal (Table 2) to work toward. Childhood obesity emerged as a primary concern for most of the groups, although each SWCC tackled this issue with a different focus. Two communities worked on the food environment, one worked on the physical environment, and four were focused on both. Many SWCCs also identified the need for better education and support for total community health.

Each community was asked to assess its own capacity and resources as part of the benchmark development process. Benchmarks ranged from conducting strategic reviews of available local physical activity resources to changing the food menus at local community events. Some SWCCs identified the need for education as paramount, while others focused on creating new or improving existing infrastructure. All communities established at least three benchmarks, and some communities established as many as six. Quantification of benchmark achievement was assessed during the follow-up interviews, and results are shown in Table 3.

### 3.1. Implementation Process Themes

Several of the key themes discussed in the follow-up interviews with SWCC leaders were related to the process in which each group employed to accomplish benchmarks and Noble Purposes. For example, community and/or stakeholder involvement was crucial to implementation of all SWCC projects. Multiple SWCCs needed to collaborate with school officials to work toward their Noble Purpose, which often involved changing school nutrition policies.

*I'm working with the food service director, the wellness coordinator, a P.E. teacher and … the health department. (Wisconsin Leader)*

Leaders talked about the benefits of working together to accomplish goals they would not have attempted or achieved individually.

*Our StrongWomen Change Club was excited and enthusiastic and wanted to be part of the change in the community. And they feel good about what they've done. You know, so that made the difference I think. (Pennsylvania Leader)*

Leaders also mentioned the waning commitment of SWCC members regarding potentially working on the project beyond the year they had committed as well as having participation gaps in the process.

*… we pulled these people from the current StrongWomen program and took them a step further in their commitment to wanting to improve the nutrition in the community. I think they've done a great job. They've stuck with us, but I also think that we did not intend to carry this project on for an extended period. You know, we told them that we were doing this for a year. (Pennsylvania Leader)*

SWCCs were interested in making the community aware of their project. Some had followed through with this, while others planned to in the future. Some SWCC enjoyed the support of local media in increasing community awareness of their SWCC's activities and goals.

### 3.2. Achievements

In the first six months, seven out of eight communities self-reported that they had successfully accomplished their first benchmark; some also reported partial success on additional benchmarks. At the 12-month assessment, all communities reported success on their first benchmark; all but one community reported at least partial success on additional benchmarks (see Table 2).

A strong theme in the post-interview was that in addition to their progress in achieving benchmarks, clubs had realized success in building awareness of the food and physical activity environments within communities.

*It had all those problems, but yet all those people came together … because of the Change Club - bringing it to their attention … we feel of anything that we've done all year long—that is our greatest accomplishment. (Alaska Leader)*

SWCC members, teachers, and children were mentioned specifically as changing or thinking about changing behaviors after becoming aware of SWCC activities and problems with the local food and physical activity environment.

*Multiple teachers have come up to me about active classrooms, about concessions, about the school wellness policy, about research on recess, so I think there's a lot more open communication and you can kind of tell the teachers are really gonna be engaged and I think I didn't have access to that before. (Wisconsin Leader)*

### 3.3. Factors That Supported and Hindered (or Slowed) Benchmark Achievement

Leaders indicated that being viewed by the community as a group that was accomplishing change helped spur further change.

*The Change Club is viewed aspeople who can get things done. (Alaska Leader)*

*Networking and that professional connection-making is huge and it takes a long time to lay that groundwork but it's hugely beneficial once you're no longer sort of just this side group, but this group that has gotten something accomplished and that has been wonderful to work with and I think that's not to be underestimated. (Alaska Leader)*

Difficulties included lack of “buy-in,” limited capacity, slow pace of community change, and waning interest of club members. Buy-in of key people in institutions or from the community in general may be necessary for successful attainment of SWCC goals. Change Clubs experienced difficulty when there was a lack of support, commitment, or interest from people or groups necessary to implement new projects.

*We had a hard time working with the food director within the school district because she was very resistant to change. (Kansas Leader)*

*I've been in city council about three times and had asked to be on a committee to discuss it and the person in charge of that said that they had bigger fish to fry. (Pennsylvania Leader)*

When there was a personnel change in key positions, new relationships had to be established before proceeding with project objectives.

*There is a new director for the program this year … So, we've kind of had to not start totally fresh, but in a sense we have. Just to kind of start our relationship with the new person and get a feel for what she would like us to do and what she does not want us to do. (Kansas Leader)*

Another barrier to successful achievement of benchmarks was the limited capacity of teachers and SWCC leaders to devote time to new projects due to existing job duties or other commitments.

*We cannot educate the teachers anyways because they're already overwhelmed. (Wisconsin Leader)*

Some Change Club leaders mentioned program implementation taking longer than they initially anticipated.

*It was like an overwhelming thing to do at first, but it's exciting and we haven't let our sight wane at all. In the next two to five years, you will be buying a plane ticket to come back (to see the project completed). (Missouri Leader)*

## 4. Discussion

The Centers for Disease Control, World Health Organization (WHO), and Institute of Medicine all recognize and recommend environmental change as a key factor in “making the healthy choice the easy choice” or the “default choice” [3, 13, 14]. The WHO recommends community participation, empowering communities to set priorities, and implements strategies to achieve better health, noting that community participation can draw on the energy and enthusiasm that exist around specific community needs [15].

The SWCC project implemented a novel approach to community-based health interventions by employing the Change Club model in seven rural communities across the United States. Local cooperative extension leaders were successful in creating groups of 8–15 women to participate in a local change project. All groups experienced a degree of benchmark success and many leaders expressed increased awareness and feelings of empowerment. However, it should be noted that during each of the initial SWCC workshops, it was clear to the research team that some groups appeared to be further along in terms of readiness to implement change. As this was not a formal component of the evaluation, no correlation between that assessment and benchmark achievement was examined, but that would be important for future studies.

Leaders that identified their benchmarks as successful or partially successful indicated that community/stakeholder engagement, SWCC dynamics, and community outreach were instrumental to success. Engagement in local politics was a recurring, key theme with leaders recognizing that key community leaders, staff, and boards (e.g., public works, school administrators) are often instrumental to implementing true socioecological changes. But such entities, or their policies and procedures, are not necessarily well-suited to rapid change. Thus, many groups found it difficult to keep members fully engaged beyond a year because of the substantial time and energy required for this work. Also, it may be valuable to establish short-term goals (e.g., start a bicycle education program) and longer-term targets (install bike route signage and roadway markings) to maintain member energy and enthusiasm beyond the initial undertaking.

There are limitations of this research that should be mentioned. First, in this project we wanted an in-depth understanding of the processes, facilitators, and barriers, and assessments were largely qualitative. Future studies should consider additional quantitative outcomes. It may be helpful to assess intermediate factors such as psychosocial markers (e.g. empowerment, self-efficacy) and whether these can be linked to community factors and related outcomes. It would be especially valuable to explore specifically how community groups can effectively influence local elected and appointed leaders, staff, and policies. However, the findings from this study can be used to help inform quantitative assessment in future studies. In addition, while this study included communities from across the U.S. and thus provides some degree of geographic diversity, the focus was on rural communities and it is a relatively small sample size. Future studies should focus on adapting the Change Club model for micropolitan and metropolitan areas. With learning from additional outcome assessments, as well as implementation of the Change Club in more diverse settings, a subsequent step will be to consider sustainability of Change Club groups and mechanisms for curriculum dissemination on a broader scale.

## 5. Conclusions

Overall, these findings are consistent with previous studies that found leveraging strong local leaders to be important to the success of health and wellness projects [16]. The barriers identified by SWCC leaders were also consistent with barriers to obesity prevention and reversal highlighted by the socioecological model, specifically social and environmental supports for behavior change [17, 18]. This project illustrates that it is possible for Change Clubs to implement local health and wellness projects based on a visioning and planning process facilitated by an academic partner.

## Figures and Tables

**Table 1:** Demographic characteristics of StrongWomen Change Club workshop participants [*N* = 107].

Characteristics	*N* (%)
Female	107 (100.00)
Married	75 (70.75)
Mean age	58.92 (range = 22–85)
Mean BMI	26.36 (range = 18–49)
Highest level of education	
High school graduate	6 (8.45)
Some college/associate's degree	28 (39.44)
Bachelor's/graduate degree	36 (50.70)
Race	
White	101 (95.28)
Hispanic	2 (1.90)
Black	1 (0.94)
Alaska native	4 (3.77)

BMI: body mass index.

**Table 2:** Noble Purposes and an example benchmark for each community [*N* = 7], 2011.

Noble Purpose	Example of benchmark
“To increase physical activity in the community using the resources we have with the goal of changing the social norms around physical activity.”	Improve signage and bridge safety for pedestrians

“To support the optimal health of children in Ouachita County by providing increased structured activity and wholesome snacks in the afterschool environment.”	Inventory and select afterschool programs for partnership

“To provide healthy food and beverages and increase time spent in active play for children in afterschool programs.”	Meet with school administration and discuss initiative

“To create and support a healthier community across generations with the goal of creating a sustainable wellness and education center that supports and is connected to other resources in town.”	Scout locations for community wellness center

“To positively shift the food culture by improving food offered at public and community gatherings.”	Refine talking points and develop a unified message

“To improve the health and wellness of school children by (1) increasing active transport to and from school and (2) implementing a healthy food environment and policy.”	Assess number of children walking and biking to school

“To foster an environment where the healthy food choice is the easy choice.”	Partner with 4–6 restaurants to offer healthy options on their menus

**Table 3:** Benchmark success assessment reported by Change Club leader (reported as percent of total participating groups)^a^.

Benchmark	Successfully accomplished^b^	Partial success	Marginal success	Did not accomplish
6-month interview				
#1	85.71%	(—)	14.29%	(—)
#2	71.43%	14.29%	(—)	14.29%
#3	57.14%	14.29%	14.29%	14.29%

12-month interview				
#1	85.71%	14.29%	(—)	(—)
#2	71.43%	14.29%	(—)	14.29%
#3	71.41%	14.29%	14.29%	(—)

^a^First three benchmarks used for standardization.

^b^Definition of “success” based on key informant's opinion.
